# Clinical management and survival outcomes of patients with different molecular subtypes of diffuse gliomas in China (2011–2017): a multicenter retrospective study from CGGA

**DOI:** 10.20892/j.issn.2095-3941.2022.0469

**Published:** 2022-11-01

**Authors:** Kenan Zhang, Xing Liu, Guanzhang Li, Xin Chang, Shouwei Li, Jing Chen, Zheng Zhao, Jiguang Wang, Tao Jiang, Ruichao Chai

**Affiliations:** 1 Department of Molecular Pathology, Beijing Neurosurgical Institute, Capital Medical University, Beijing 100070, China; 2 Department of Neuropathology, Beijing Neurosurgical Institute, Capital Medical University, Beijing 100070, China; 3 Department of Neurosurgery, Beijing Tiantan Hospital, Capital Medical University, Beijing 100070, China; 4 Department of Neurosurgery, Beijing Sanbo Brain Hospital, Capital Medical University, Beijing 100093, China; 5 Division of Life Science and State Key Laboratory of Molecular Neuroscience, Department of Chemical and Biological Engineering, The Hong Kong University of Science and Technology, Clear Water Bay, Kowloon, Hong Kong SAR 999077, China; 6 Hong Kong Center for Neurodegenerative Diseases, Hong Kong Science Park, Hong Kong SAR 999077, China; 7 HKUST Shenzhen-Hong Kong Collaborative Innovation Research Institute, Futian, Shenzhen 518057, China

**Keywords:** Diffuse glioma, IDH, 1p/19q, molecular pathology, temozolomide

## Abstract

**Objective:**

We aimed to summarize the clinicopathological characteristics and prognostic features of various molecular subtypes of diffuse gliomas (DGs) in the Chinese population.

**Methods:**

In total, 1,418 patients diagnosed with DG between 2011 and 2017 were classified into 5 molecular subtypes according to the 2016 WHO classification of central nervous system tumors. The IDH mutation status was determined by immunohistochemistry and/or DNA sequencing, and 1p/19q codeletion was detected with fluorescence in situ hybridization. The median clinical follow-up time was 1,076 days. T-tests and chi-square tests were used to compare clinicopathological characteristics. Kaplan-Meier and Cox regression methods were used to evaluate prognostic factors.

**Results:**

Our cohort included 15.5% lower-grade gliomas, IDH-mutant and 1p/19q-codeleted (LGG-IDHm-1p/19q); 18.1% lower-grade gliomas, IDH-mutant (LGG-IDHm); 13.1% lower-grade gliomas, IDH-wildtype (LGG-IDHwt); 36.1% glioblastoma, IDH-wildtype (GBM-IDHwt); and 17.2% glioblastoma, IDH-mutant (GBM-IDHm). Approximately 63.3% of the enrolled primary gliomas, and the median overall survival times for LGG-IDHm, LGG-IDHwt, GBM-IDHwt, and GBM-IDHm subtypes were 75.97, 34.47, 11.57, and 15.17 months, respectively. The 5-year survival rate of LGG-IDHm-1p/19q was 76.54%. We observed a significant association between high resection rate and favorable survival outcomes across all subtypes of primary tumors. We also observed a significant role of chemotherapy in prolonging overall survival for GBM-IDHwt and GBM-IDHm, and in prolonging post-relapse survival for the 2 recurrent GBM subtypes.

**Conclusions:**

By controlling for molecular subtypes, we found that resection rate and chemotherapy were 2 prognostic factors associated with survival outcomes in a Chinese cohort with DG.

## Introduction

Diffuse glioma (DG) frequently leads to severe consequences, including death and disability. DG accounts for more than 80% of primary malignancies in the central nervous system (CNS)^1,2^. Each year, more than 30,000 patients are diagnosed with DG in China^3–5^. Despite comprehensive treatment including surgery, chemotherapy, radiotherapy (RT), and tumor treating fields, the overall survival (OS) of patients varies substantially, ranging from half a year to more than 10 years^6,7^.

Accurate diagnosis and classification are essential for improving the clinical management of DG^8,9^. Traditional methods based on histological appearance and immunohistochemical staining for protein expression remain insufficient for patient classification and precise management. In the past 2 decades, neuropathologists have grouped tumors according to genetic changes, identified hundreds of molecular biomarkers, and gradually revised the classification to include diagnostic categories based on genotypes^10–12^. In the 5th edition of the WHO classification of CNS tumors published in 2021, more molecular features were established as diagnostic criteria for molecular subtypes. However, the surgery, postsurgical treatment, and prognosis of Chinese patients with DG in each subgroup—classified according to integrated diagnosis based on histological features and molecular features, *IDH1/2* mutation, and chromosome 1p/19q codeletion included in the 2016 WHO classification—remain largely unclear^13^.

On the basis of the WHO 2016 classification of CNS tumors, DGs were graded from II to IV. Because grade II and III glioma commonly share similar genetic alterations, DGs are also commonly classified into lower-grade glioma (LGG) and glioblastoma (GBM)^14,15^, and then classified into 5 subgroups: LGG IDH-mutant and 1p/19-codeleted (LGG-IDHm-1p/19q); LGG IDH-mutant without 1p/19q codeletion (LGG-IDHm); LGG IDH-wildtype (LGG-IDHwt); GBM IDH-wildtype (GBM-IDHwt); and GBM IDH-mutant (GBM-IDHm)^16–18^. An American multicenter cohort study has revealed different ages at diagnosis and OS between subgroups^19^. Recent studies have shown that the responses to similar treatment strategies differ between subgroups^20,21^, and surgical strategies should differ according to subgroups^22–24^. However, owing to the lag between clinical practice and pathological classification guidelines, particularly the imbalance of diagnostic levels in China, large-scale cohort studies designed to systematically reveal the clinicopathological features, survival outcomes, prognostic factors, and responses to therapies in different subgroups of the Chinese population with DG remain lacking.

Our previous study analyzed these factors in patients with glioma with a traditional histological classification strategy, and described the prognostic roles of several immunohistochemically tested markers, e.g., *TP53* and *Ki-67*^6,25^. Here, we retrospectively analyzed patients from 2011 to 2017 included in the Chinese Glioma Genome Atlas (CGGA) project from 3 major neurosurgical centers, constituting the largest Chinese cohort with DG^26^. We aimed to determine the survival outcomes, clinicopathological features, prognostic factors, and treatment benefits of the different subgroups according to the WHO 2016 classification, thus providing national reference data for the improvement and development of clinical treatment guidelines in China.

## Materials and methods

### Patient inclusion

The study included all patients who underwent surgical resection and were diagnosed with DG at Beijing Tiantan Hospital, Beijing Puren Hospital, and Beijing Sanbo Brain Hospital from January 2011 to December 2017. All participants were consistently diagnosed with glioma by 2 independent neuropathologists. All studies performed were approved by the Institutional Review Board (IRB) of Beijing Tiantan Hospital (IRB: KY2013-017-01) and were conducted according to the principles of the Declaration of Helsinki. Written informed consent was obtained from all patients.

### Clinicopathological information

Clinical data were collected from the medical records of patients, which included sex, age at diagnosis, pre- and post-operative Karnofsky performance scores (KPS scores), symptom at onset, tumor location, extent of resection, histological type, radiotherapy, and temozolomide (TMZ) treatment information. The exact tumor location was assessed with preoperative MRI by experienced neurosurgeons. The histological diagnosis was double-checked by 2 independent neuropathologists, and patients were further categorized according to the 2007 or 2016 WHO classification in different periods.

Molecular neuropathological information was collected from the hospital information system. The IDH mutation status was tested by sequencing or immunohistochemistry (IHC). For patients diagnosed before 2016, the IDH mutant information was first detected with IHC staining with an antibody to IDH1 R132H; we also retested the IDH1 R132 and IDH2 R172 hotspot status in younger patients (< 65 years old) with a negative IDH1 IHC result by performing pyrosequencing. For patients diagnosed between 2016 and 2017, IDH mutation information was obtained directly from IDH1 R132 and IDH2 R172 hotspot pyrosequencing. Chromosome 1p/19q deletion was detected with fluorescence in situ hybridization. MGMT promoter methylation was tested with pyrosequencing^8,27^.

### Treatment

The extent of resection was assessed by 2 independent experienced radiologists with MRI images captured within 2 weeks of resection^28^. Total resection, subtotal resection, major partial resection, and partial resection were defined as none, nodular or thin, less than half, or more than half residual T2 or FLAIR signal abnormalities. Patients who received radiotherapy or TMZ refer to those receiving an entire treatment course^2,29^.

### Follow-up

Survival information was collected through telephone interviews. Death and malignant progression were confirmed through follow-up. Patient recovery performance, post-surgery RT, and chemotherapy treatment information were also collected. OS was calculated from the day of the surgery to the date of death or the end of follow-up, and progression-free survival (PFS) was defined as the period between the day of surgery and radiographic progression (the appearance of a new lesion or an increase in the residual tumor size by more than one-quarter)^22,24,25^. Overall, the median follow-up time of all enrolled patients was 1,076 days.

### Statistical analysis

All analyses and visualizations were performed with the R package (V4.1.0). T tests and chi-squared tests were used to determine differences between variables. The Kaplan-Meier method was used to analyze survival data with the R packages “survival” and “survminer.” Cox analysis (backward) was performed in SPSS V26 for Windows (SPSS Inc., Chicago, IL, USA). Variants with *P* values < 0.1 in the univariate Cox analysis were included in the multivariate Cox analysis^19,30^. A two-sided *P* value of 0.05 was considered statistically significant.

## Results

### Patient characteristics

**Figure 1:**
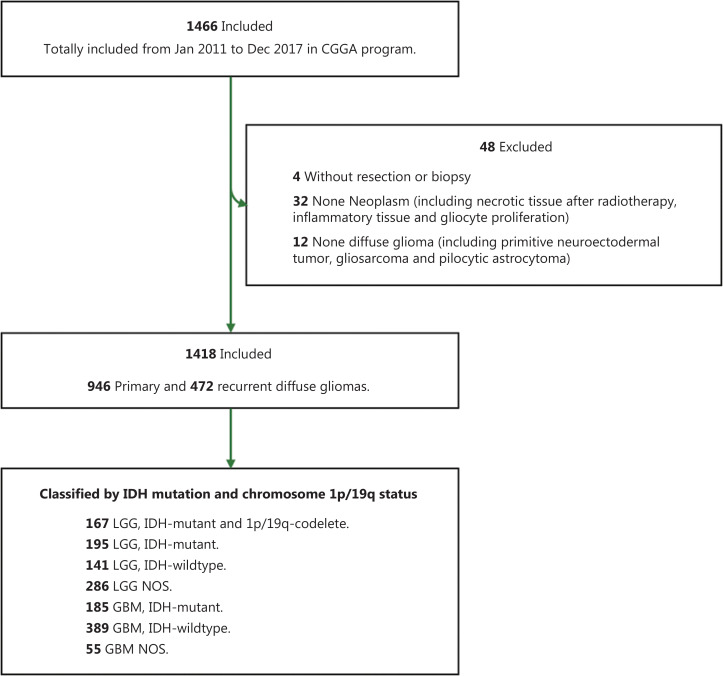
Flowchart of patients with eligible diffuse gliomas who were included in the study.

In total, 1,466 patients who were diagnosed with DG on the basis of MRI features were collected from 3 medical centers. After the exclusion of patients who did not undergo surgical resection or were not pathologically diagnosed with DG, 1,418 were included in the present study (**Figure 1**). Additionally, all patients with a history of cancers except DGs were excluded. Patient characteristics are summarized in **Table 1**, including sex, age, clinical manifestations, tumor location, histological grade, and molecular subtype. Overall, 839 men with a mean age of 43.4±12.5 years and 579 women with a mean age of 43.7±12.3 years were included. A total of 946 cases (66.7%) were primary DGs, whereas 472 (33.3%) were recurrent tumors. Regarding symptoms at diagnosis, we observed 555 patients (41.6%) with headache, 457 (34.3%) with seizures, 393 (29.4%) with neurofunctional deficits, and 179 (13.4%) without clear symptoms. Among the enrolled patients, most had gliomas in the frontal lobe (67.0%); some had tumors in the temporal lobe (42.2%), insular lobe (22.2%), or parietal lobe (21.4%); and few had tumors in the occipital lobe (8.6%) or other cortical regions (5.2%).

**Table 1:** Distribution of 1418 cases of diffuse glioma according to clinicopathological information

Characteristics	Diffuse glioma	Primary diffuse glioma	Recurrent diffuse glioma	*P*
Sex				
Male	839	541	298	0.0339
Female	579	405	174	
Age				
≥ 45	659	460	200	0.0276
< 45	755	486	272	
Mean age, years (SD)	43.55 (12.33)	44.33 (12.95)	42.00 (10.67)	
Symptom at onset (*n* = 1334)				
Headache	555	445	110	< 0.0001
Seizure	457	331	126	
Focal deficit	393	240	153	
No clear symptoms	179	58	121	
Karnofsky performance status (*n* = 1330)				
≥ 80	1260	857	403	< 0.0001
< 80	70	30	40	
Lateral involvement (*n* = 1390)				
Right	656	450	206	0.6024
Left	659	439	220	
Both sides	70	43	27	
Midline	5	3	2	
Cortex involvement (*n* = 1390)				
Frontal lobe	931	607	324	0.0017
Temporal lobe	586	386	200	
Insular lobe	309	237	72	
Parietal lobe	298	183	115	
Occipital lobe	120	77	43	
Other lobes	72	50	22	
Histological grade				
II	376	309	67	< 0.0001
III	413	290	123	
IV	629	347	282	
Molecular subtype				
LGG, IDH-mutant and 1p/19q-codeleted	167	130	37	< 0.0001
LGG, IDH-mutant	195	130	65	
LGG, IDH-wildtype	141	103	38	
LGG, NOS	286	236	50	
GBM, IDH-wildtype	389	248	141	
GBM, IDH-mutant	185	63	122	
GBM, NOS	55	36	19	
MGMT methylation (*n* = 1080)				
Unmethylated	374	251	123	0.4077
Methylated	706	492	214	
Types of surgery				
Total resection	627	475	152	< 0.0001
Subtotal resection	511	333	178	
Partial resection	278	138	140	
Biopsy	2	0	2	
Treatment (*n* = 1274)				
Radio chemotherapy	643	520	123	< 0.0001
Radiotherapy	173	151	22	
Chemotherapy	263	74	189	
None	195	111	84	

The distribution of proportions of patients stratified by sex, age at diagnosis, symptom at onset, KPS, tumor location, histological grade, and molecular subtype (according to subgroups based on the WHO 2016 classification) significantly differed between patients with primary and recurrent DGs (**Table 1**). As expected, larger proportions of patients with an older age at diagnosis, grade IV tumors, and GBM-IDHm were diagnosed with recurrent tumors. However, no laterality or difference in MGMT methylation status was observed between patients with primary and recurrent DGs.

### Clinical management

For more than 15 years, the standard treatment for patients with DG has been surgical resection followed by radiotherapy and/or treatment with the DNA-alkylating agent TMZ, as recommended by clinical guidelines^2,29,31^. In our cohort, 1,138 patients underwent total (44.2%) or subtotal (36.0%) resection, 278 patients (19.6%) underwent partial resection, and 2 patients (1.4‰) underwent biopsy (**Table 1**). With the use of TMZ oral agents, more patients received standard and effective chemotherapy, including 50.5% of patients who received standard RT with concurrent or adjuvant TMZ chemotherapy and 20.6% of patients who received TMZ chemotherapy alone. Additionally, 13.5% of patients received only standard RT. Meanwhile, 15.3% of patients did not receive any radio- or chemotherapy.

The selection of treatment strategies was also quite different between patients with primary and recurrent DGs (**Table 1**). A larger proportion of patients with primary tumors underwent total resection than did patients with recurrent tumors (50.2% *vs*. 32.2%, *P* < 0.0001). Meanwhile, more patients with recurrent tumors (40.0% *vs*. 7.8%, *P* < 0.0001) received only chemotherapy, because patients who received RT at the initial diagnosis were not advised to receive RT again.

### Molecular classification and subtype characteristics

On the basis of the WHO 2016 classification of CNS tumors, 946 primary DGs were classified into 5 molecular subtypes through the integrated diagnosis of histological features and the status of *IDH1/2* mutation and chromosome 1p/19q deletion (**Table 2**). Consequently, 130 patients were classified into the LGG-IDHm-1p/19q subtype, 130 were classified into the LGG-IDHm subtype, 103 were classified into the LGG-IDHwt subtype, 248 were classified into the GBM-IDH-wt subtype, and 63 were classified into the GBM-IDHm subtype in our cohort. The remaining patients were classified as LGG, not otherwise specified (LGG-NOS, *n* = 236), owing to the lack of information on 1p/19q deletion status, or GBM-NOS (*n* = 36), owing to the lack of information on *IDH1/2* mutation status. As expected, relatively more (*P* < 0.0001) patients were older at diagnosis in the GBM-IDH-wt subgroup. The diagnostic symptom of neurofunctional deficit was also present in a relatively higher proportion of patients (*P* < 0.0001) in the GBM-IDH-wt subgroup. Interestingly, the distributions of patients with lateral involvement (*P* = 0.0408) and cortical region involvement (*P* < 0.0001) also significantly differed among molecular subtypes.

**Table 2:** Characteristics of primary diffuse gliomas

	*n*	LGG, IDH-mutant and 1p/19q-codeleted	LGG, IDH-mutant	LGG, IDH-wildtype	GBM, IDH-wildtype	GBM, IDH-mutant	*P*	LGG NOS	GBM NOS
Sex	946	130	130	103	248	63	0.7995	236	36
Male	541	72	75	59	150	40	0.7995	123	22
Female	405	58	55	44	98	23		113	14
Age									
≥ 45	460	56	37	34	195	28	< 0.0001	83	27
< 45	486	74	93	69	53	35		153	9
Mean age, years	44.30	42.15	39.15	38.55	52.86	43.67		41.17	49.75
SD of age	12.73	9.89	8.57	16.04	12.57	11.79		9.98	11.57
Karnofsky performance status									
≥ 80	857	126	123	100	233	58	0.3722	189	28
< 80	30	4	3	2	14	2		4	1
Diagnostic symptom	890	130	127	102	247	62	< 0.0001	193	29
Headache	445	49	56	48	156	43		77	16
Seizure	331	68	64	40	37	18		95	9
Neurofunction deficit	240	22	19	29	114	12		35	9
No clear symptom	58	15	6	8	5	1		22	1
Lateral involved	935	130	129	101	244	63	0.0408	233	35
Right	450	66	66	44	110	29		114	21
Left	439	57	57	46	123	32		113	11
Both sides	43	7	6	8	11	2		6	3
Midline	3	0	0	3	0	0		0	0
Cortex involved (*n*)	935	130	129	101	244	63	< 0.0001	233	35
Frontal lobe	607	103	93	51	114	43		181	22
Temporal lobe	386	40	50	40	118	32		92	14
Insular lobe	237	34	44	21	46	16		73	3
Parietal lobe	183	17	21	22	67	11		34	11
Occipital lobe	77	1	5	7	45	5		13	1
Other lobes	50	6	6	10	19	3		6	0

For patients with recurrent tumors, 37 LGG-IDHm-1p/19qs, 65 LGG-IDHms, 38 LGG-IDHwts, 141 GBM-IDHwts, and 122 GBM-IDHms were identified. Compared with patients with primary tumors, patients with recurrent DGs had a markedly greater proportion of GBM-IDHm (*P* < 0.0001, **Table 1**), thus implying the malignant progression of LGG-IDHm to GBM-IDHm.

### Clinical follow-up for primary DGs

**Figure 2:**
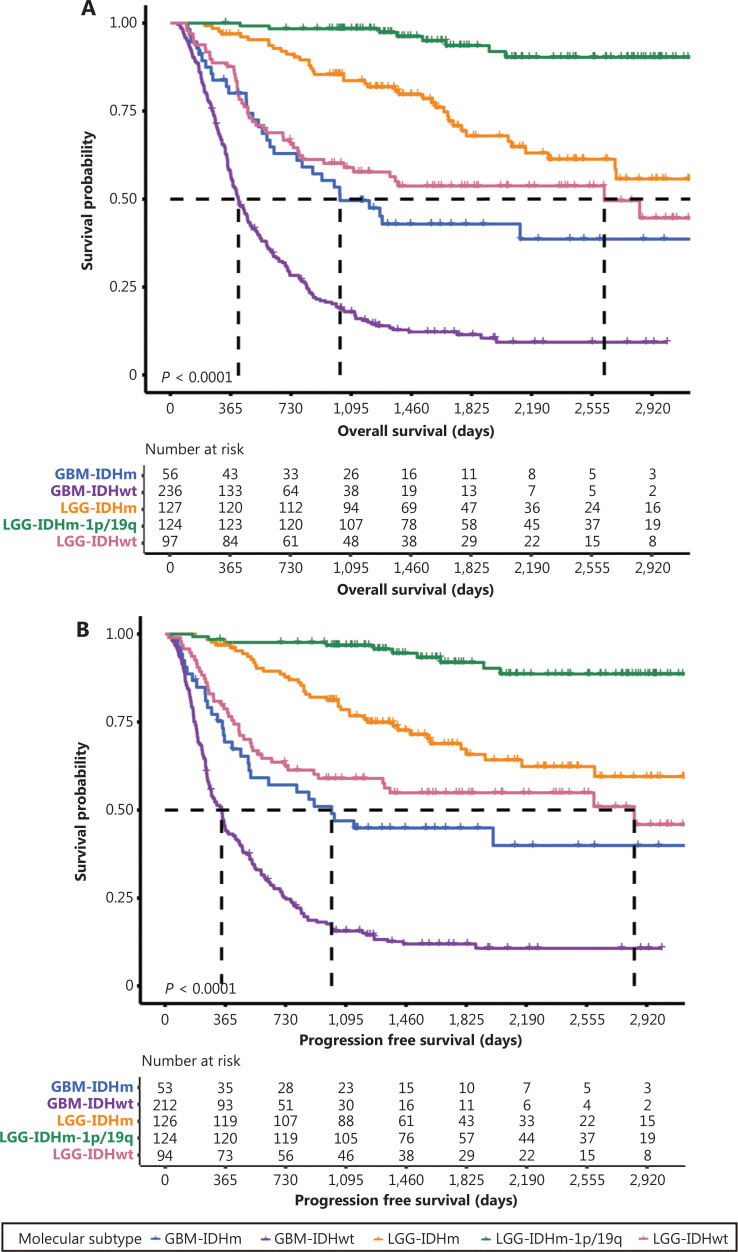

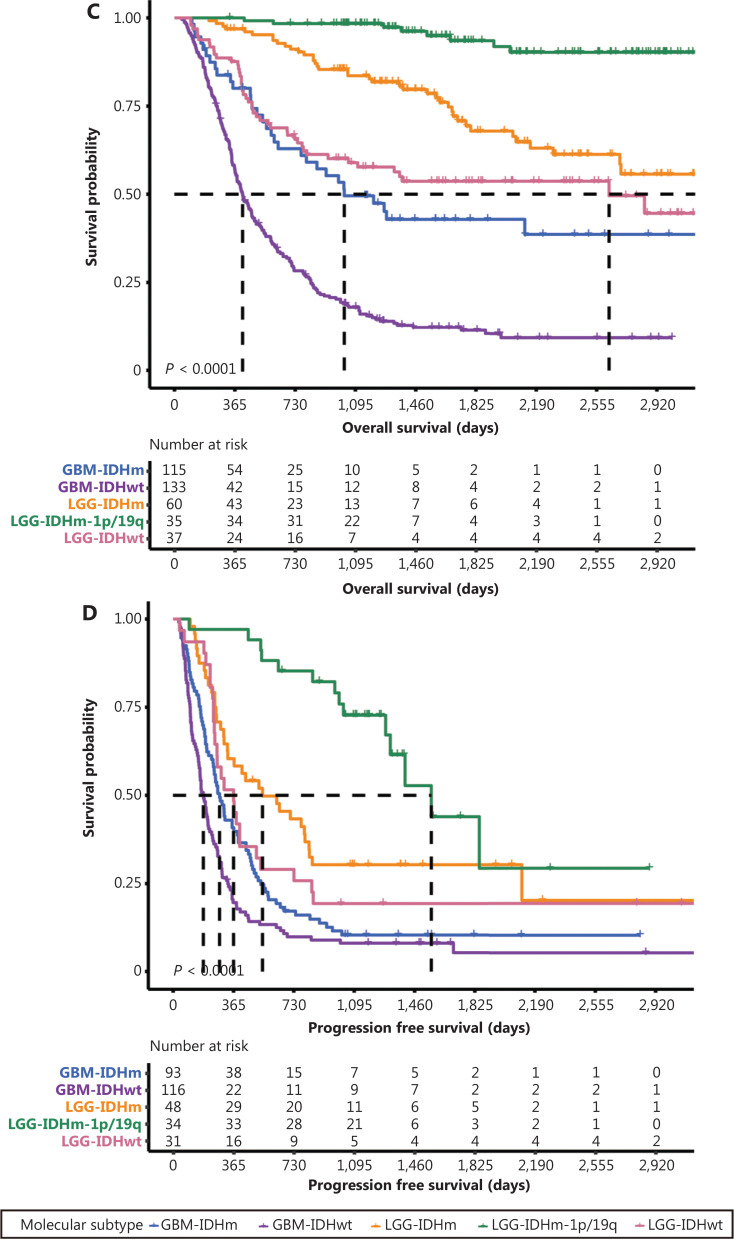
Survival outcomes of patients with different molecular subtypes. Kaplan-Meier estimates of overall survival (A) and progression-free survival (B) of patients with primary DG classified according to molecular subtypes. Kaplan-Meier estimation of the overall survival (C) and progression-free survival (D) of patients with recurrent DG classified according to molecular subtypes.

Follow-up information was available for 640 patients with primary DGs. Kaplan-Meier estimates of OS and PFS according to tumor molecular subtypes are shown in **Figure 2**. The median OS of patients with GBM-IDHwt, GBM-IDHm, LGG-IDHwt, and LGG-IDHm was 11.57, 15.17, 34.57, and 75.97 months, respectively (**Figure 2A**). The median PFS of patients with GBM-IDHwt, GBM-IDHm, LGG-IDHwt, and LGG-IDHm was 8.63, 12.97, 30.03, and 86.8 months, respectively (**Figure 2B**). The 5-year survival rate for patients with LGG-IDHm-1p/19q was 76.54%, and less than half the patients with oligodendroglioma in our cohort experienced recurrence or died (**Supplementary Table S1**). The OS and PFS rates of patients with each molecular subtype and histological grade were also summarized (**Supplementary Tables S1 and S2**).

For patients with recurrent DGs, the median OS of the GBM-IDHwt, GBM-IDHm, LGG-IDHwt, LGG-IDHm, and LGG-IDHm-1p/19q subgroups was 8.53, 11.87, 17.03, 18.77, and 52.93 months, respectively (**Figure 2C**). The median PFS of patients with GBM-IDHwt, GBM-IDHm, LGG-IDHwt, LGG-IDHm, and LGG-IDHm-1p/19q was 6.07, 9.33, 12.0, 18.0 and 52.03 months, respectively (**Figure 2D**).

### Prognostic factors for primary DGs

Because we had only post relapse survival data available for patients with recurrent tumors, we focused on identifying the prognostic factors for primary DGs. We conducted univariate and multivariate Cox regression analyses and found that the age at diagnosis, histological grade, molecular subtype, post-surgery KPS, resection rate, and chemotherapy were significantly associated with OS in multivariate Cox analyses of all patients with DGs (all *P* < 0.05, **Supplementary Table S3**). No significant association between pre-surgery KPS and patient OS was observed in this analysis, owing to the strong correlation between pre- and post-surgery KPS, thus implying the importance of preserving brain function during surgery. Interestingly, the univariate Cox regression analysis revealed that men were more at risk than women (*P* = 0.014).

We further analyzed prognostic factors for each molecular subtype. The histological grade and resection rate were significantly correlated with OS in patients with all 3 LGG subtypes. Post-surgery KPS was significantly correlated with OS in patients with LGG-IDHwt. Neither radiotherapy nor chemotherapy significantly correlated with OS in patients with LGGs (**Table 3**). For patients with GBMs, a higher resection rate (*P* value < 0.05 for both GBM-IDHwt and GBM-IDHm) and treatment with chemotherapy (*P* value < 0.05 for GBM-IDHwt, and *P* value = 0.02 for GBM-IDHm) were significantly associated with better prognosis in both the IDH-wildtype and IDH-mutant subgroups. Older age (*P* = 0.061) and lower post-surgery KPS (*P* = 0.003) were associated with shorter OS in patients with GBM and IDHwt (**Table 4**).

**Table 3:** Univariate and multivariate Cox proportional-hazards models for low grade gliomas

Variable	Hazard ratio (95% CI)					
	LGG, IDH-mutant and 1p/19q-codeleted		LGG, IDH-mutant		LGG, IDH-wildtype	
	Univariate	Multivariate	Univariate	Multivariate	Univariate	Multivariate
Age at diagnosis^1^	1.069 (0.998–1.144)^†^	1.378 (1.08–1.759)^*^	0.984 (0.948–1.021)		1.026 (1.007–1.045)^†^	
Sex						
Female	Reference		Reference		Reference	
Male	0.702 (0.175–2.811)		1.331 (0.684–2.591)		1.598 (0.867–2.944)	
Histological grade^2^	5.84 (0.713–47.86)^†^	48.808 (1.193–1997.239)^*^	3.902 (1.789–8.51)^†^	2.659 (1.195–5.918)^*^	3.776 (1.808–7.887)	6.024 (2.311–15.702)^*^
MGMT						
Unmethylated	Reference		Reference		Reference	
Methylated	2.657 (0.318–22.229)		0.566 (0.284–1.125)		0.376 (0.194–0.729)^†^	
KPS_PRE^3^	0.991 (0.885–1.109)		0.98 (0.922–1.041)		0.971 (0.931–1.013)	
KPS_POST^4^	0.925 (0.869–0.986)^†^		0.933 (0.903–0.964)^†^	0.956 (0.925–0.988)^*^	0.944 (0.92–0.969)^†^	0.949 (0.906–0.994)^*^
Resection rate						
Total resection	Reference	Reference	Reference	Reference	Reference	Reference
Subtotal resection	4.68 (0.424–51.663)	24.532 (0.522–1153.043)	2.083 (0.911–4.765)^†^	1.707 (0.724–4.025)	6.022 (1.368–26.507)^†^	11.326 (1.407–91.164)^*^
Major partial resection	147.156 (13.035–1661.254)^†^	4288.619 (17.419–1.056^10^6^)^*^	21.3 (8.518–53.262)^†^	13.381 (5.079–35.251)^*^	28.795 (6.524–127.102)^†^	46.648 (5.71–381.063)^*^
Partial resection	251.105 (17.041–3700.168)^†^	615281.86 (102.016–3.711^10^9^)^*^	253.199 (43.115–1486.938)^†^	120.991 (17.383–842.133)^*^	42.932 (9.598–192.039)^†^	100.392 (11.692–861.985)^*^
Radiotherapy						
Not received	Reference		Reference		Reference	
Received	1.386 (0.279–6.894)		1.1 (0.459–2.636)		1.292 (0.616–2.708)	
Chemotherapy						
Not received	Reference		Reference		Reference	
Received	1.585 (0.318–7.902)		0.748 (0.373–1.503)		2.011 (0.924–4.373)^†^	

^1^The hazard ratio is for each 1-yr increase in age. ^2^The hazard ratio is for each 1 grade increase in WHO grade. ^3^The hazard ratio is for each 1 point increase in KPS score. ^4^The hazard ratio is for each 1 point increase in KPS score. ^†^The *P* value of the hazard ratio was less than 0.1 for the univariate Cox models and included in the multivariate Cox model. ^*^The hazard ratio was significant (*P* < 0.05).

**Table 4:** Univariate and multivariate cox proportional-hazards models for glioblastomas

Variable	Hazard ratio (95% CI)			
	Glioblastoma, IDH-wildtype		Glioblastoma, IDH-mutant	
	Univariate	Multivariate	Univariate	Multivariate
Age at diagnosis^1^	1.018 (1.007–1.03)^†^	1.012 (0.999–1.025)	1.012 (0.985–1.041)	
Sex				
Female	Reference		Reference	
Male	1.052 (0.794–1.393)		1.208 (0.584–2.499)	
MGMT				
Unmethylated	Reference		Reference	
Methylated	0.938 (0.706–1.246)		0.858 (0.424–1.739)	
KPS_PRE^2^	0.987 (0.967–1.007)		0.96 (0.908–1.015)	
KPS_POST^3^	0.962 (0.949–0.975)^†^	0.975 (0.959–0.991)^*^	0.965 (0.934–0.998)^†^	
Resection rate				
Total resection	Reference	Reference	Reference	Reference
Subtotal resection	2.269 (1.646–3.127)^†^	2.074 (1.468–2.929)^*^	1.814 (0.804–4.093)	1.771 (0.726–4.319)
Major partial resection	7.259 (4.677–11.267)^†^	5.598 (3.469–9.033)^*^	30.002 (6.037–149.107)^†^	66.577 (9.7–456.942)^*^
Partial resection	84.175 (41.344–171.378)^†^	76.094 (30.649–188.918)^*^	99.335 (20.047–492.219)^†^	361.066 (37.041–3519.607)^*^
Radiotherapy				
Not received	Reference		Reference	
Received	0.582 (0.396–0.854)^†^		0.771 (0.315–1.888)	
Chemotherapy				
Not received	Reference	Reference	Reference	Reference
Received	0.297 (0.198–0.447)^†^	0.42 (0.271–0.652)^*^	0.279 (0.113–0.691)^†^	0.309 (0.115–0.829)^*^

^1^The hazard ratio is for each 1-yr increase in age. ^2^The hazard ratio is for each 1 point increase in KPS score. ^3^The hazard ratio is for each 1 point increase in KPS score. ^†^The *P* value of the hazard ratio was less than 0.1 for the univariate Cox models and included in the multivariate Cox model.^*^The hazard ratio was significant (*P* < 0.05).

### Therapeutic response to comprehensive treatment

**Figure 3:**
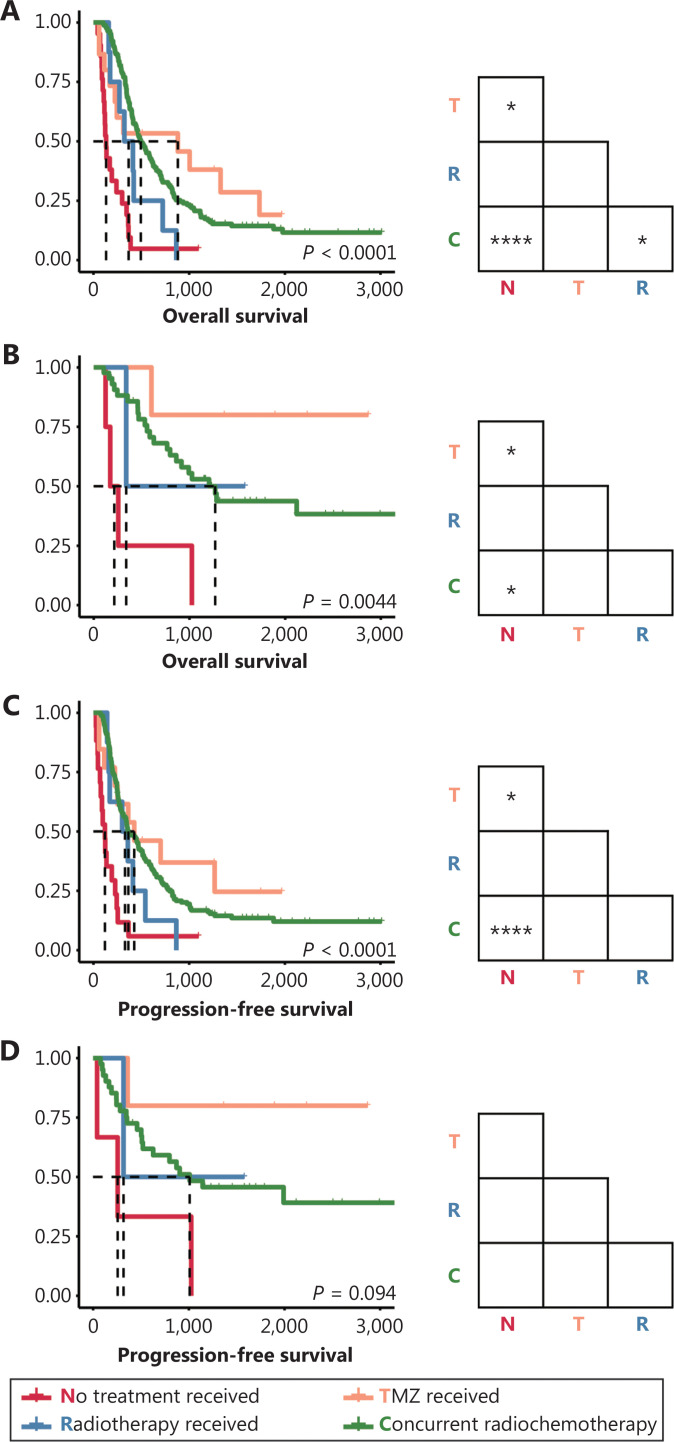
Survival outcomes of patients with primary GBM receiving different postsurgical treatments. (A–B) Kaplan-Meier curves estimating the overall survival of patients with primary GBM-IDHwt (A) and patients with primary GBM-IDHm (B). (C–D) Kaplan-Meier curves estimating the progression-free survival of patients with primary GBM-IDHwt (C) and patients with primary GBM-IDHm (D). *P < 0.05, ****P < 0.0001.

We also compared the survival of patients with each molecular subtype who received different treatments, to further explore the responses of tumors to radiotherapy and/or chemotherapy. Consequently, both patients with IDH-wild-type and IDH-mutant GBM who received TMZ and/or RT had longer OS than did patients who did not receive these treatments (**Figure 3A, 3B**). Meanwhile, PFS was prolonged in patients with GBM-IDHwt who received TMZ and/or RT but not in patients with GBM-IDHm (**Figure 3C, 3D**). In agreement with the results of the Cox analysis (**Table 3**), nonsignificant differences in OS and PFS were observed in patients with different postsurgical treatments among all 3 subgroups of LGG (**Supplementary Figure S1**).

**Figure 4:**
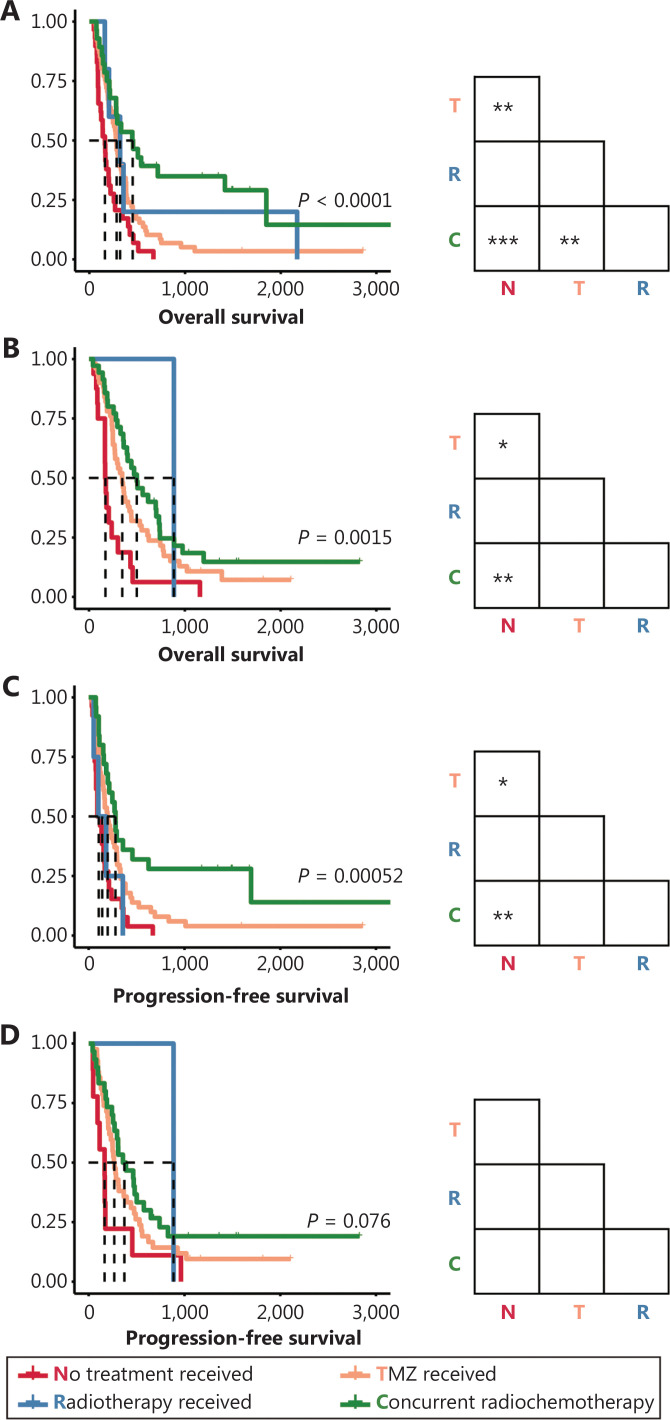
Survival outcomes of patients with recurrent GBM receiving different postsurgical treatments. (A–B) Kaplan-Meier curves estimating overall survival of patients with recurrent GBM-IDHwt patients (A) and patients with recurrent GBM-IDHm (B). (C–D) Kaplan-Meier curves estimating progression-free survival of patients with recurrent GBM-IDHwt (C) and patients with recurrent GBM-IDHm (D). *P < 0.05, **P < 0.01, ***P < 0.001.

Interestingly, chemotherapy or concurrent radio-chemotherapy also prolonged the post-relapse OS and PFS of patients with recurrent tumors (**Figure 4**). TMZ (*P* < 0.01) and RT with TMZ (*P* < 0.001) significantly prolonged the post-relapse OS of patients with recurrent GBM-IDHwt (**Figure 4A**). Similarly, TMZ (*P* < 0.05) and RT with TMZ (*P* < 0.01) significantly prolonged the post-relapse PFS in patients with recurrence (**Figure 4C**). Meanwhile, OS in patients with GBM-IDHm was also prolonged by TMZ (*P* < 0.05) and concurrent radio-chemotherapy (*P* < 0.01) (**Figure 4B**). A similar trend was also observed in the PFS of these patients, and the nonsignificant differences among groups may be attributable to the insufficient cohort size (**Figure 4D**).

## Discussion

DG accounts for most primary intracranial malignancies^32^. The prognosis of patients with different histological types varies substantially. A simple classification based on morphological characteristics is not sufficiently reliable to categorize patients into clinically and etiologically homogeneous groups. Owing to extensive efforts to profile genomic characteristics, rapid advances in the molecular pathology of DG have been reported in recent years^33^. Mutations of *IDH1/2* and codeletion of chromosomal 1p/19q were first introduced into the WHO classification of CNS tumors. Many studies have suggested that treatment strategies should be guided by the different molecular subtypes^34,35^. Recently, the molecular classification has also been used by the National Comprehensive Cancer Network (NCCN) clinical practice guidelines for CNS cancers (Version 1.2022, June 2, 2022). However, large Chinese cohort studies evaluating the clinicopathological feature distribution, prognosis, and treatment responses of patients with DGs in the molecular era remain lacking. Here, we analyzed patients in the CGGA project collected from 3 major neurosurgical medical centers from 2011 to 2017, thus revealing the clinical management and survival status of patients with DGs in each subgroup classified according to an integrated diagnosis of histological and molecular (IDH mutant and 1p/19q co-deleted) features. Compared with our previous findings^6^, the survival of patients with LGG was improved in this period: the 3-year survival rate of patients with WHO grade II and grade III tumors increased from 79% in 2004–2010 to 86% in this cohort and 51% in 2004–2010 to 62% in this cohort, respectively. A similar trend was also observed for the 5-year survival rate, which increased from 67% to 68% and 36% to 44% for patients with WHO grade II and grade III tumors, respectively. However, the changes in the survival of patients with GBM remain uncertain, because the 3-year survival rate increased from 15% to 17%, whereas the 5-year survival rate decreased from 9% to 6%. The survival of our patients was not inferior to that in other non-Chinese cohorts from developed countries, such as the United Kingdom, Japan, and the United States; moreover, patients with GBM survived even longer^36–39^.

Notably, the present study revealed that surgical resection played a crucial role in determining patient prognosis. The resection rate significantly correlated with the survival outcomes of patients with all molecular subtypes. New strategies are being used to improve the protection efficiency, such as awake craniotomy, neuro-navigation, intraoperative MRI, 5-aminolevulinic acid, and techniques that do not involve labeling^40,41^. Controversy persists regarding whether total resection is beneficial for patients with LGGs, particularly for tumors that invade the functional cortex^42,43^. Our results included the postoperative KPS rather than the preoperative KPS in the multivariate Cox regression model. This analysis implied the importance of protecting brain function during tumor resection surgery^44^.

In our study, subtotal resection, compared with total resection, was not a significant factor in the outcomes of patients with IDH-mutant molecular subtypes, thereby indicating that the extent of resection exerts different effects on the prognosis of patients with different molecular subtypes^23,45^. Different resection strategies should be considered for patients with different molecular subtypes. Likewise, some studies have recommended less resection combined with effective comprehensive treatment for LGG, which may improve patient survival and quality of life^24,46,47^.

Radio-genomics, along with machine learning methods, is a promising approach to acquire molecular information before resection^48–50^. Our previous studies have verified the feasibility of predicting molecular information for IDH mutation, TP53 mutation, and chromosome 1p/19q codeletion^51–53^. Thus, surgical strategies may be tailored according to molecular subtype.

In this study, compared with our previous studies, more patients received chemo- and/or radiotherapy treatment after surgical resection^6,25^, thus suggesting an improvement in the postsurgical management of patients in China. Overall, the more aggressive and effective treatment extended both the OS and PFS of patients, although the effectiveness of chemotherapy varied among molecular subtypes. At present, the effect of chemotherapy on LGG remains an important open issue^54,55^. Patients with the 3 LGG subtypes in our cohort did not benefit from radio- or chemotherapy.

Interestingly, sex was also significantly associated with patient survival in specific molecular groups in our cohort, e.g., GBM-IDHwt. To our knowledge, this study is the first to reveal the prognostic role of sex in a Chinese cohort, although similar results have been reported in Western cohorts^56–59^. Several explanations have been suggested, including hormone rhythms, lifestyle, psychological status, and genetic inheritance^60,61^, and women appear to have a stronger protective response against DGs.

Two limitations of this study must be noted. The molecular characteristics of a small portion of patients were missing because molecular testing only gradually began to be performed in recent years. Thus, one-quarter of patients were diagnosed with NOS^62^. Another limitation is that the follow-up time for some patients with LGGs remains insufficient. Consequently, the median overall survival of patients diagnosed with LGG-IDHm-1p/19q was not available in this study.

Updates to the Consortium to Inform Molecular and Practical Approaches to CNS Tumor Taxonomy (cIMPACT-NOW) and the 2021 fifth edition of the WHO classification of CNS tumors have been published^63^. Increasing numbers of genetic alterations are being included to classify or grade DGs more precisely^64,65^. The entity of GBM-IDHm was substituted by a novel subtype of astrocytoma, CNS WHO grade 4. However, the effects of key molecules included in the WHO 2016 Classification on clinical management have not been sufficiently evaluated in large clinical cohorts of Chinese patients, thus reflecting the lag in clinical application of the pathological classification guidelines. Our findings provide basic reference data for analyzing the effects of the WHO 2016 guidelines on the clinical management of glioma in the Chinese population. We will include additional molecular pathological information in the CGGA cohort to evaluate the effects of the WHO 2021 classifications on Chinese patients in the near future.

## Conclusions

In conclusion, by conducting the largest multicenter analysis of the management of patients with DG in China to date, we demonstrated the general survival outcomes of patients with DG. Chinese patients with different subtypes of DG based on integrated diagnosis with the WHO 2016 classification have distinct clinicopathological features, survival, prognostic factors, and responses to radiotherapy and/or chemotherapy. Our study suggests that the updated WHO classification scheme should be adapted in clinical management and clinical trials as soon as possible.
