# Erratum to Clinical management and survival outcomes of patients with different molecular subtypes of diffuse gliomas in China (2011–2017): a multicenter retrospective study from CGGA

**DOI:** 10.20892/j.issn.2095-3941.2023.0227

**Published:** 2023-07-24

**Authors:** Kenan Zhang, Xing Liu, Guanzhang Li, Xin Chang, Shouwei Li, Jing Chen, Zheng Zhao, Jiguang Wang, Tao Jiang, Ruichao Chai

**Affiliations:** 1 Department of Molecular Pathology, Beijing Neurosurgical Institute, Capital Medical University, Beijing 100070, China; 2 Department of Neuropathology, Beijing Neurosurgical Institute, Capital Medical University, Beijing 100070, China; 3 Department of Neurosurgery, Beijing Tiantan Hospital, Capital Medical University, Beijing 100070, China; 4 Department of Neurosurgery, Beijing Sanbo Brain Hospital, Capital Medical University, Beijing 100093, China; 5 Division of Life Science and State Key Laboratory of Molecular Neuroscience, Department of Chemical and Biological Engineering, The Hong Kong University of Science and Technology, Clear Water Bay, Kowloon, Hong Kong SAR 999077, China; 6 Hong Kong Center for Neurodegenerative Diseases, Hong Kong Science Park, Hong Kong SAR 999077, China; 7 HKUST Shenzhen-Hong Kong Collaborative Innovation Research Institute, Futian, Shenzhen 518057, China

In the published version of **Figure 2**^1^, an error appeared in **Figure 2C** on page 1468. In **Figure 2C**, the Kaplan-Meier estimation of the overall survival of patients with recurrent DG classified according to molecular subtypes was mistakenly covered by the curves of patients with primary DGs during the figure layout process, while the number statistic under the figure is correct. **Figure 2C** has been updated to correct this mistake. The error does not affect the conclusions of this article. We apologize for the error and for any confusion that it might have caused.

**Figure 2C:**
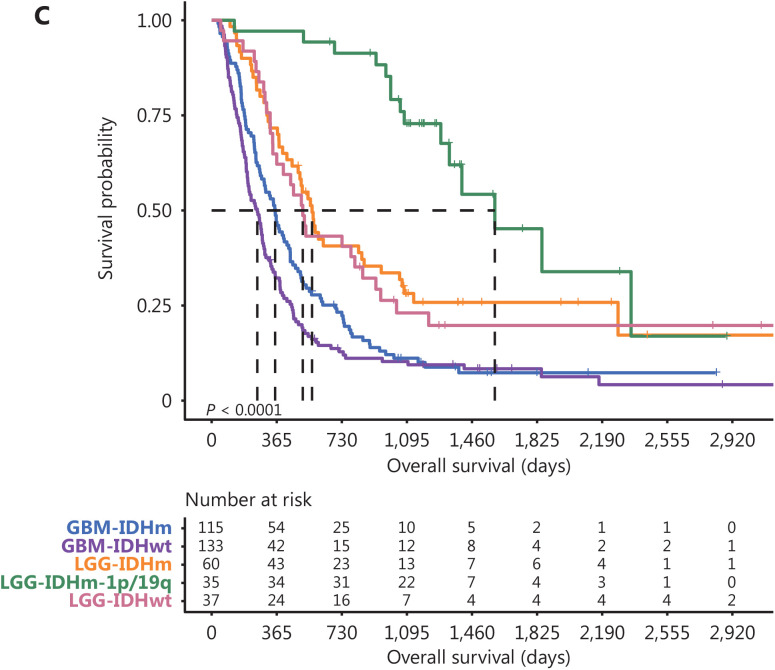
Kaplan-Meier estimation of the overall survival of patients with recurrent DG classified according to molecular subtypes.
