# Supplementary material for: Clinical management and survival outcomes of patients with different molecular subtypes of diffuse gliomas in China (2011–2017): a multicenter retrospective study from CGGA
Source: Cancer Biol Med. 2022 Nov 1;19(10):1460–76. doi: 10.20892/j.issn.2095-3941.2022.0469 (PMC9630520; doi:10.20892/j.issn.2095-3941.2022.0469)
Supplement: Supplementary file 1 [file cbm-19-1460-s001.pdf]

Supplementary materials

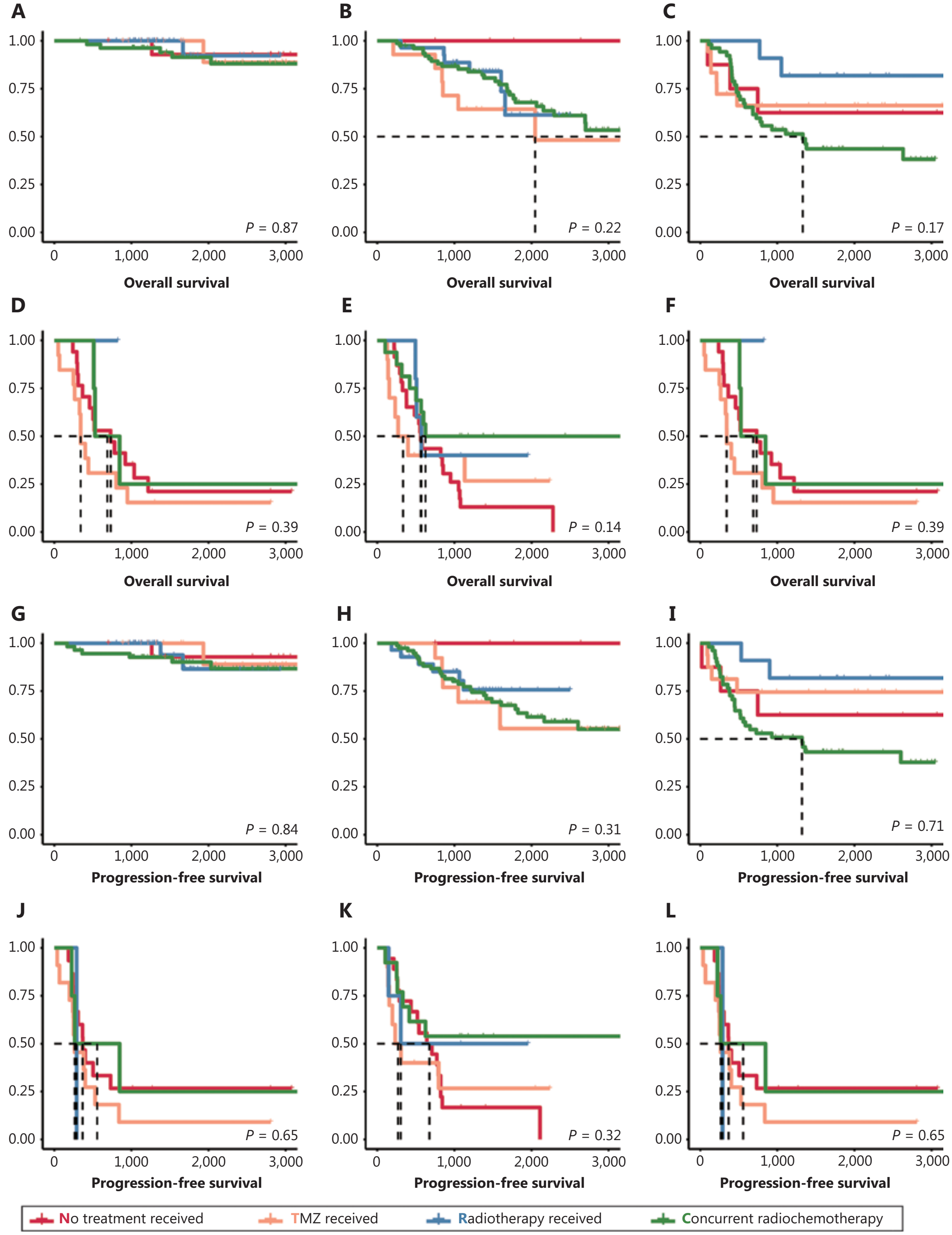

Figure S1 Survival outcomes of patients with LGG receiving different postsurgical treatments. (A–C) Kaplan–Meier curves estimating the overall survival of patients with primary LGG-IDHm-1p/19q (A), LGG-IDHm (B), and LGG-IDHwt (C). (D–F) Kaplan–Meier curves estimating the overall survival of patients with recurrent LGG-IDHm-1p/19q (D), LGG-IDHm (E), and LGG-IDHwt (F). (G–I) Kaplan–Meier curves estimating progression-free survival of patients with primary LGG-IDHm-1p/19q (G), LGG-IDHm (H), and LGG-IDHwt (I). (J–L) Kaplan–Meier curves estimating progression-free survival of patients with recurrent LGG-IDHm-1p/19q (J), LGG-IDHm (K), and LGG-IDHwt (L).

Table S1 Overall survival (OS) and progression-free survival (PFS) in patients with diffuse gliomas

	LGG, IDH-mutant and 1p/19q-codeleted	LGG, IDH-mutant	LGG, IDH-wildtype	GBM, IDH-mutant	GBM, IDH-wildtype
Overall survival rate (%)					
At 1 year	99.37	88.59	81.20	57.40	47.81
At 3 year	92.14	63.31	45.08	21.95	13.97
At 5 year	76.54	41.09	31.43	8.84	5.01
Progression free survival rate (%)					
At 1 year	96.84	88.62	72.36	50.69	35.60
At 3 year	90.65	62.66	45.13	21.43	12.23
At 5 year	74.07	40.67		9.76	4.33

Table S2 Overall survival (OS) and progression-free survival (PFS) in patients with diffuse gliomas grouped by WHO grade

WHO histological grade	II	III	IV
Median OS (month)	N/A	55.63	12.53
OS rate (%)			
At 1 year	96.86	89.09	52.41
At 3 year	86.27	61.64	16.84
At 5 year	67.60	43.52	6.12
Median PFS (month)	N/A	53.07	9.5
PFS rate (%)			
At 1 year	95.32	85.07	41.10
At 3 year	86.38	59.48	15.83
At 5 year	67.80	43.55	5.90

Table S3 Univariate and multivariate Cox proportional-hazards models for primary diffuse gliomas

Variable	Hazard ratio (95% CI)	
	Univariate	Multivariate
Age at diagnosis[1]	1.051 (1.042–1.06)	1.014 (1.004–1.024)
Sex		
Female	Reference	
Male	1.285 (1.049–1.575)	
Histological grade[2]	3.98 (3.392–4.669)	3.624 (2.233–5.884)
Molecular subtype		
LGG, IDH-mutant and 1p/19q-codeleted	Reference	Reference
LGG, IDH-mutant	5.375 (2.516–11.484)	5.998 (2.626–13.699)
LGG, IDH-wildtype	10.202 (4.808–21.647)	6.113 (2.69–13.893)
LGG, NOS	3.09 (1.449–6.589)	3.545 (1.517–8.286)
GBM, IDH-mutant	14.461 (6.676–31.325)	4.239 (1.523–11.796)
GBM, IDH-wildtype	36.718 (18.055–74.67)	10.304 (3.904–27.19)
GBM, NOS	23.821 (10.63–53.381)	3.641 (0.829–15.993)
MGMT		
Unmethylated	Reference	
Methylated	0.498 (0.401–0.618)	
KPS_pre[3]	0.945 (0.931–0.959)	
KPS_post[4]	0.937 (0.928–0.945)	0.971 (0.958–0.984)
Resection rate		
Total resection		
Subtotal resection	2.142 (1.68–2.73)	2.499 (1.878–3.325)
Major partial resection	8.397 (6.289–11.211)	9.957 (6.928–13.311)
Partial resection	16.44 (11.752–22.997)	51.13 (30.918–84.557)
Radiotherapy		
Not received	Reference	
Received	1.286 (0.978–1.691)	
Chemotherapy		
Not received	Reference	Reference
Received	1.897 (1.459–2.467)	0.603 (0.443–0.822)

[1]The hazard ratio is for each 1-yr increase in age. [2]The hazard ratio is for each 1 grade increase in WHO grade. [3]The hazard ratio is for each 1 point increase in KPS score. [4]The hazard ratio is for each 1 point increase in KPS score.
